# The extrafascicular phloem is made for fighting

**DOI:** 10.3389/fpls.2013.00187

**Published:** 2013-06-11

**Authors:** Frank Gaupels, Andrea Ghirardo

**Affiliations:** ^1^Helmholtz Zentrum München, German Research Center for Environmental Health, Department of Environmental Sciences, Institute of Biochemical Plant PathologyNeuherberg, Germany; ^2^Helmholtz Zentrum München, German Research Center for Environmental Health, Department of Environmental Sciences, Institute of Biochemical Plant Pathology, Research Unit Environmental SimulationNeuherberg, Germany

The fascicular (bundle) phloem (FP) distributes assimilates from photosynthetic active source leaves to sinks such as young leaves, meristems and roots. It is also involved in long-distance signaling and defence responses (Van Bel and Gaupels, 2004; Walz et al., 2004; Lough and Lucas, 2006). In the past, cucurbits were frequently used as model plants for phloem biochemistry because large quantities of phloem exudates can be easily sampled from incisions into petioles and stems. Analysing pumpkin (*Cucurbita maxima*) exudates more than 1100 phloem proteins and about 100 metabolites could be identified providing some insight into phloem functions (Fiehn, 2003; Lin et al., 2009).

However, recent publications convincingly demonstrated that cucurbit exudates do not represent pure FP sap but rather originate mainly from the extrafascicular phloem (EFP) blended with xylem fluid (Zhang et al., 2010, 2012; Gaupels et al., 2012; Zimmermann et al., 2013). The EFP is a network of sieve tubes outside of the vascular bundles found only in *Cucurbitaceae*. Due to this unique characteristic, knowledge obtained from analyses of EFP exudates must be carefully verified to apply also for the FP of other plants (Turgeon and Oparka, 2010).

These findings settled a long-standing debate on the origin and purity of cucurbit phloem exudates, but one important question is still not resolved: what is actually the main function of EFP and EFP-derived exudates in cucurbits? We will discuss here the hypothesis that the EFP functions in herbivore and pathogen defence similar to laticifers in other plant species. Consequently, for better differentiation from FP sap and owing to the latex-like properties EFP exudates will be termed phloem latex hereafter.

## Extrafascicular phloem vs. fascicular phloem

The EFP consists of a complex network of perifascicular strands next to the vascular bundles, lateral commissural strands, entocyclic sieve tubes within the pit and ectocyclic sieve tubes in the cortex (Crafts, 1932). The companion cell/sieve element complexes of the EFP strands are similar in shape and diameter to the FP (Golecki et al., 1999). However, absence of the EFP from minor veins of source leaves suggests that this particular phloem is not involved in sugar loading (Turgeon and Oparka, 2010). The fact that the EFP does not connect sink and source tissues would generally argue against an important role of the EFP in assimilate distribution.

A detailed analysis of the sugar composition revealed low sugar concentrations in phloem latex due to dilution of EFP content with xylem fluid (Zhang et al., 2012; Zimmermann et al., 2013). A similar dilution effect was also observed for FP exudates suggesting similar sugar concentration and osmotic pressure within both phloem systems. However, the sugar composition differed considerably between FP and the various elements of the EFP. In the perifascicular phloem—like in the FP—the transport sugars stachyose and sucrose were most abundant whereas in ento- and ectocyclic sieve tubes non-mobile hexoses were most prominent (Zhang et al., 2012). These findings indicate that the perifascicular but not other elements of the EFP contribute to assimilate transport. Further studies e.g., using ^13^C- or ^14^C-labeling techniques (Ghirardo et al., 2011; Zhang et al., 2012) are needed to gain more detailed information of assimilate transport in the EFP.

Notably, the different types of extrafascicular sieve tubes are all involved in transport processes as evidenced by translocation of the phloem-mobile tracer 5(6)-carboxyfluorescein (Zhang et al., 2010) and graft-transmission of several phloem proteins including the major Phloem protein1 (PP1) and Phloem protein2 (PP2) (Golecki et al., 1999) within the EFP. PP1 and PP2 were also immuno-localized in the FP although the corresponding genes were mainly expressed in companion cells of the EFP, which would hint at plasmodesmal connections between the two phloem systems of cucurbits (Golecki et al., 1999).

Apart from symplasmic continuity, the ~50% overlap of so-far identified FP proteins from rice (*Oryza sativa*), rape (*Brassica napus*) and castor bean (*Ricinus communis*) with phloem latex proteins from pumpkin also suggested some degree of functional similarity between EFP and FP (Lin et al., 2009). On the other hand, the same comparison revealed that the EFP contained the complete machinery for protein translation, which is not present in the FP probably as an adaptation to assimilate transport functions (Lough and Lucas, 2006; Lin et al., 2009). Even within one plant—namely pumpkin—the protein composition was found to be disparate between exudates from EFP and FP (Zhang et al., 2010; Gaupels et al., 2012). Particularly, PP1 and PP2 make up to 80% of total protein content in phloem latex but were not detected by 1- and 2-dimensional polyacrylamid gel electrophoresis in FP sap of pumpkin.

In sum, the discussed data support the notion that EFP and FP are physically and functionally connected. This applies particularly to the perifascicular sieve tubes of the EFP. Given the reported differences in structure as well as sugar and protein composition it seems, however, likely that the EFP network is at least partially separated from FP and probably has other functions than assimilate transport.

## Extrafascicular phloem vs. laticifers

Laticifers are specialized cells forming tubular systems with a distinct cytoplasmic content known as latex. A proposed function of laticifers is the synthesis and storage of compounds involved in herbivore and pathogen defence (Hagel et al., 2008; Konno, 2011). Here, we define latex as a plant exudate from intracellular stores with primarily defensive functions (Konno, 2011), which may but must not contain rubber particles (but cf. Pickard, 2008). Eminent examples of economically relevant latex-producing plants are opium poppy (*Papaver somniferum*) and para rubber tree (*Hevea brasiliensis*). Depending on the species laticifers can originiate from various cell types. Interestingly, anastomosing (net-like) laticifers develop from phloem initials and are tightly associated with the vascular tissue (Hagel et al., 2008). Here, we hypothesize that the anastomosing EFP system shares common functions with anastomosing laticifers.

An important feature of laticifers is the secretion of copious amounts of latex from wounds. This way, insects are confronted with large droplets of the sticky and toxic fluid. Similarly, cucurbits bleed profoundly upon wounding. The exudation is driven by the high osmotic pressure in the EFP and by diffusion of xylem water into the EFP after wound-induced pressure release (Zhang et al., 2012; Zimmermann et al., 2013). The EFP content was estimated to be ~100-fold diluted by xylem fluid (Zhang et al., 2012). If the FP, which is rapidly plugged by callose, contributes considerably to phloem latex is still ambiguous and might vary between species (Zhang et al., 2012).

Previously, the EFP was assumed to be devoid of efficient sieve tube plugging by callose (Turgeon and Oparka, 2010; Zhang et al., 2012). However, wound-inducible polysaccharide synthesis reminiscent of callose formation (Gaupels et al., 2012) as well as SIEVE ELEMENT OCCLUSION proteins (Lin et al., 2009) were detected in pumpkin phloem latex. These findings would imply that EFP occlusion is delayed or suppressed for facilitating unrestricted exudation from cuts. Only after unloading of the phloem latex the occlusion mechanisms are probably essential for reestablishment of the EFPs osmotic pressure and defensive arsenal (Gaupels et al., 2012).

Callose or other carbohydrates and proteins such as PP1 and PP2 which coagulate upon exudation could have dual functions (1) by causing the observed stickiness of phloem latex (Gaupels et al., 2012), which is essential for clogging insect mouth parts as was reported for the defence strategy of cucurbits against squash beetle (*Epilachna borealis*) (McCloud et al., 1995). (2) Additionally, callose and PP1/PP2 polymers could be involved in covering the wound site for sealing and protection from microbial ingress into the vascular system (Read and Northcote, 1983; Turgeon and Oparka, 2010).

**Figure 1:**
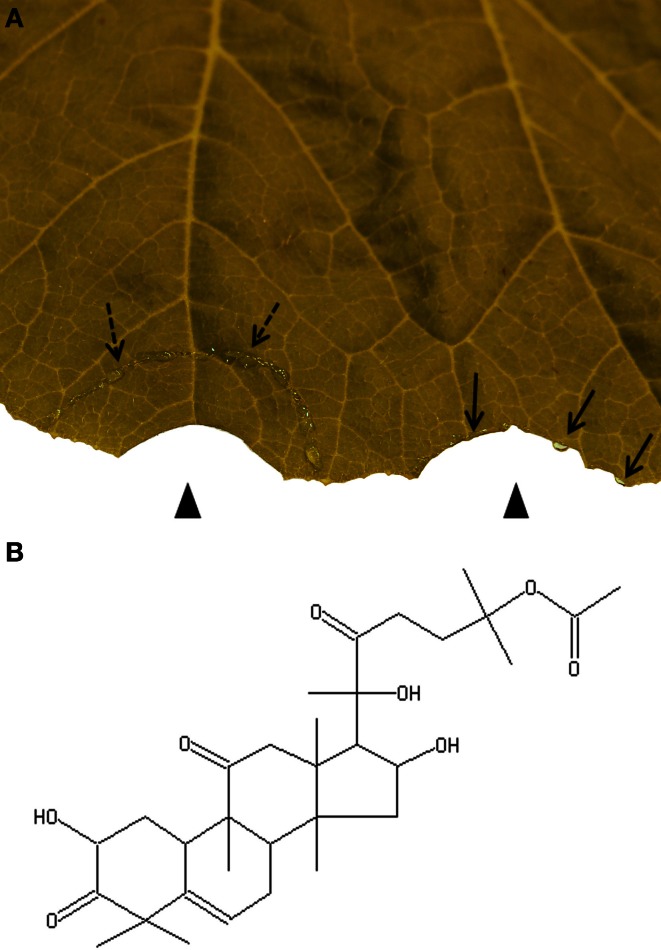
**Trenching is a strategy of herbivorous insects for avoiding ingestion of toxic phloem latex. (A)** Artificial trenching releases phloem latex (broken arrows). The isolated semi-circular area is cleared from phloem latex as demonstrated by a cut from which no exudate emerges (left arrow head). In contrast droplets of exudates (arrows) appear from a cut outside of the trenched area (right arrow head). **(B)** Cucurbitacin B is present in most cucurbits.

The pressure-driven exudation of both laticifers and EFP can be circumvented by trenching (Figure 1A). Some beetle species isolate a circular leaf area through cutting all tissues except for the lower epidermis. Through this adaptive behavior the beetles clear the feeding area from harmful latex (Carroll and Hoffman, 1980; Tallamy, 1985; Konno, 2011).

## Defence-related molecules in phloem latex

To date, cucurbitacins are the best studied defensive metabolites in cucurbit phloem latex. They constitute a heterologous family of tetracyclic triterpenoids with a bitter taste and high cytotoxicity (Chen et al., 2005). Some cucurbitacins display antibacterial and antifungal activities. Others were shown to be involved in insect defence by acting as an antifeedant or antagonizing the effect of insect steroid hormones (Chen et al., 2005). For instance, when host leaves were painted with cucurbitacin B (Figure 1B) all six tested species of non-specialist herbivorous insects were deterred from feeding and two species were deterred from oviposition (Tallamy et al., 1997). However, specialist feeders of cucurbits can tolerate cucurbitacins or even use them for their own defence system (Carroll and Hoffman, 1980; Agrawal et al., 1999). Cucurbitacins are constitutively present in phloem latex but levels further increase locally and systemically after herbivore attack (Carroll and Hoffman, 1980; Tallamy, 1985; Agrawal et al., 1999).

Inducible defence responses against herbivores and certain pathogens are under control of the plant hormone jasmonic acid (JA). After leaf wounding JA and its bioactive conjugate JA-isoleucine rapidly accumulated within 30 min in pumpkin phloem latex collected from distant petioles and stems indicating the onset of a systemic wound response (SWR) (Gaupels et al., 2012). During SWR JA is synthesized in the phloem and is transported as a systemic phloem-mobile signal (Li et al., 2002; Gaupels et al., 2012; Gaupels and Vlot, 2012).

Downstream-targets of JA in the EFP remain to be elucidated but could include amongst others the phloem latex proteins SILVERLEAF-WHITEFLY-INDUCED PROTEIN1, 18-kD CYCLOPHILIN, 16-kD PHLOEM PROTEIN1 and MITOGEN-ACTIVATED PROTEIN KINASE6, which were all increased in protein level after leaf damage (Gaupels et al., 2012). Further abundant proteins in cucurbit exudates are elements of the constitutive defence such as peroxidases, proteinases as well as PHLOEM SERPIN1 and other proteinase inhibitors (Walz et al., 2004; Frohlich et al., 2012; Gaupels et al., 2012). Similar defence proteins are also widespread in latex from laticifers (Konno, 2011).

The most remarkable protein in phloem latex is PP2. This protein has several proposed functions in defence and signaling. First of all, PP2 is a lectin. AtPP2-A1—the closest Arabidopsis homolog of cucurbit PP2—was shown to bind N-acetylglucosamine and glycans (Beneteau et al., 2010). The corresponding gene is inducible by the bacterial elicitor hairpin and transgenic overexpression of AtPP2-A1 induced resistance against the aphid *Myzus persicae* without exact mechanisms of resistance known (Zhang et al., 2011). Hence, PP2 could be involved both in defence against bacterial pathogens and phloem-sucking insects. Moreover, upon exposure to air oxygen the redox-sensitive PP2 and PP1 are responsible for stickiness and gelation of phloem latex as a defence trait against herbivorous beetles (McCloud et al., 1995).

PP2 and PP1 interact via intermolecular disulfide bridges between cysteine residues forming insoluble gel-like polymers under oxidizing conditions (Read and Northcote, 1983). However, even under non-stress conditions PP1 and PP2 self-assemble to filaments in the EFP while only a small proportion of the proteins is mobile (Smith et al., 1987; Golecki et al., 1999). At high levels mobile PP1 and PP2 would probably interfere with assimilate transport and therefore, filaments are stored in the EFP until pressure-released from cuts. After leaf wounding pumpkin PP2 abundance decreased transiently concomitant with a decline in protein carbonylation/oxidation suggesting that PP2 might be redox-modified under stress conditions (Gaupels et al., 2012). This redox-modification might trigger a monomerization and mobilization of PP2 in the phloem. PP2 monomers could act as defensive lectins or carriers of mRNA signals like recently shown in melon (*Cucumis melo*) (Gomez et al., 2005; Beneteau et al., 2010).

Other defensive proteins in phloem latex typically also found in latex from other plants include a large set of protease inhibitors, proteases, peroxidases, and lipoxygenase (Walz et al., 2004; Konno, 2011). Although not directly related to herbivore defence it is significant that both in latex from *Hevea brasiliensis* as well as in pumpkin phloem latex, proteins of the translation and proteasome complexes including ribosomal proteins, eukaryotic translation initiation factors and elements of the proteasome constitute major functional categories whereas these proteins are largely missing in the FP (Lin et al., 2009; D'Amato et al., 2010; Frohlich et al., 2012; Gaupels et al., 2012). We speculate here that this finding reflects the special laticifer-like functions of the EFP, which necessitate extensive biosynthesis of defensive proteins and enzymes involved in the production of secondary metabolites.

In sum, the accumulation of cucurbitacins and major proteins related to signaling and defence responses in phloem latex further supports a protective role of the EFP system against herbivore attack and subsequent microbial infection.

## Conclusions

The main function of phloem is distributing assimilates. Because the phloem contains highly nutritive molecules, which appeal insects and pathogens, it was evolutionary forced to develop efficient defence measures. In cucurbits the tasks of transporting assimilates and defending against attackers are shared by two specialized phloem systems. The FP is a linear and simply branched tubular system optimized for unhindered sugar translocation. In contrast, the structure of the EFP is net-like for better coverage of all tissues and improved resistance against insect counter-defences e.g., by vein cutting. The content of the EFP is highly enriched in proteins (PP1/PP2) and compounds with viscous, sticky and toxic properties. These features all resemble laticifers and would severly interfere with assimilate transport functions of phloem. Collective evidence rather supports the view that the EFP acts similar to laticifers as a pressure-driven defence mechanism against insects and pathogens.
